# Elastography in Reproductive Medicine, a Game-Changer for Diagnosing Polycystic Ovary Syndrome, Predicting Intrauterine Insemination Success, and Enhancing In Vitro Fertilization Outcomes: A Systematic Review

**DOI:** 10.3390/biomedicines13040784

**Published:** 2025-03-24

**Authors:** Charalampos Voros, Antonia Varthaliti, Despoina Mavrogianni, Diamantis Athanasiou, Antonia Athanasiou, Aikaterini Athanasiou, Anthi-Maria Papahliou, Constantinos G. Zografos, Vasileios Topalis, Panagiota Kondili, Menelaos Darlas, Sophia Sina, Maria Anastasia Daskalaki, Panagiotis Antsaklis, Dimitrios Loutradis, Georgios Daskalakis

**Affiliations:** 1 1st Department of Obstetrics and Gynecology, ‘Alexandra’ General Hospital, National and Kapodistrian University of Athens, 80 VasilissisSofias Avenue, 11528 Athens, Greece; antonia.varthaliti@hotmail.com (A.V.); depy.mavrogianni@yahoo.com (D.M.); anthipapahliou@gmail.com (A.-M.P.); giotakondyli27@gmail.com (P.K.); mdarlas2110@gmail.com (M.D.); sophia_sina@yahoo.com (S.S.); md181341@students.euc.ac.cy (M.A.D.); panosant@gmail.com (P.A.); gdaskalakis@yahoo.com (G.D.); 2 IVF Athens Reproduction Center V. Athanasiou, 15123 Maroussi, Greece; diamathan16@gmail.com (D.A.); antoathan16@gmail.com (A.A.); aikathanasiou@hotmail.com (A.A.); 3 2nd Surgical Department, General Hospital of Athens “LAIKO”, 11527 Athens, Greece; koszogra92@hotmail.com; 4 Department of Internal Medicine, Hospital of Thun, 3600 Thun, Switzerland; vtopalismd@gmail.com; 5 Fertility Institute-Assisted Reproduction Unit, Paster 15, 11528 Athens, Greece; loutradi@otenet.gr; 6 Athens Medical School, National and Kapodistrian University of Athens, 15772 Athens, Greece

**Keywords:** elastography, polycystic ovary syndrome, intrauterine insemination, in vitro fertilization, endometrial receptivity, reproductive medicine, shear wave elastography, real-time elastography, infertility diagnostics

## Abstract

**Background**: Elastography is an ultrasound-based imaging technology that allows for quantitative measurement of tissue stiffness and elasticity. In reproductive medicine, it is a potential non-invasive method for assessing ovarian activity, uterine contractility, and endometrial receptivity. While conventional ultrasound provides anatomical and vascular information, it does not assess biomechanical properties, which are important for understanding polycystic ovary syndrome (PCOS), predicting intrauterine insemination (IUI) success, and determining endometrial receptivity in in vitro fertilization (IVF). **Methods**: A systematic review was conducted in accordance with the PRISMA principles, and the protocol was recorded in PROSPERO. A comprehensive literature search was conducted across several databases to uncover studies that used real-time elastography (RTE) or shear wave elastography (SWE) for PCOS diagnosis, IUI result prediction, or endometrial receptivity evaluation in IVF. The risk of bias was assessed using the ROBINS-I technique. **Results**: Four studies fulfilled the inclusion criteria. One study indicated that PCOS patients had considerably increased ovarian stiffness, which supports elastography as a diagnostic marker. Another study found that increased uterine flexibility and decreased contractility were related with better IUI outcomes. A retrospective cohort research discovered that non-uniform endometrial echogenicity had no influence on IVF results. Furthermore, SWE successfully evaluated endometrial receptivity in unexplained infertility, with higher stiffness being related to reduced implantation potential. **Conclusions**: Elastography gives real-time, quantitative insights into reproductive biomechanics, with potential applications in infertility diagnosis and ART improvement. However, the absence of defined imaging procedures and confirmed clinical criteria prevent its broad use. More large-scale prospective investigations are required to improve elastographic parameters and define diagnostic cutoffs for clinical use.

## 1. Introduction

Infertility is a complicated medical problem that affects millions of couples worldwide, with an estimated frequency of 10–15% among people of reproductive age [1]. Despite significant advances in assisted reproductive technology, many instances remain unexplained, and accurately assessing ovarian activity, uterine contractility, and endometrial receptivity remains a difficulty in clinical practice [2]. The efficacy of fertility therapies like intrauterine insemination and in vitro fertilization is dependent on precise diagnostic instruments that can examine the reproductive environment holistically [3]. While transvaginal ultrasonography and Doppler imaging continue to be the primary procedures for assessing ovarian and endometrial morphology, they give only limited quantitative data on tissue biomechanical properties [4]. The discovery of elastography, a unique ultrasound-based imaging technology that measures tissue stiffness and elasticity, has opened up new possibilities in reproductive medicine by providing a more thorough and objective assessment of reproductive tissues [5].

Elastography, which has been widely employed in hepatology and cancer, is currently being investigated for its potential use in gynecology and reproductive health. This imaging technique examines the mechanical characteristics of tissues in real time, allowing for the diagnosis of abnormalities that may not be visible with conventional ultrasonography [6].

Elastography can be carried out using two ultrasound-based techniques: real-time elastography (RTE) and shear wave elastography (SWE). Real-time elastography (RTE) is a qualitative or semi-quantitative method for measuring tissue stiffness that employs external compression via an ultrasonic probe and operator-dependent manual pressure. Tissue elasticity is represented by color-coded pictures, whereas stiffness is portrayed by strain ratios that compare target tissue with nearby normal reference tissue. While RTE gives useful qualitative information about tissue stiffness, its reliance on manual compression presents possible unpredictability and operator dependence. Shear wave elastography (SWE), on the other hand, quantifies tissue stiffness by creating acoustic shear waves within the tissue using focused ultrasonic pulses, eliminating the need for physical compression. SWE objectively assesses tissue stiffness by estimating the propagation speed of these shear waves (m/s), which is directly proportional to tissue elasticity (kPa). SWE outperforms RTE in terms of reproducibility and accuracy due to its quantitative nature and lower operator dependency. Clinically, both techniques offer advantages and disadvantages. RTE is especially beneficial for initial qualitative assessment and rapid screening in gynecological disorders, including ovarian abnormalities in PCOS, because qualitative stiffness patterns can swiftly reveal altered ovarian morphology. SWE, on the other hand, is typically used for more exact, quantitative measurements—especially in situations requiring reliable assessment and monitoring of endometrial receptivity in IVF or predicting uterine elasticity for IUI success. As a result, SWE is widely regarded as more reliable and reproducible for precise clinical decision-making and longitudinal monitoring, but RTE is still useful for initial diagnostic tests.

Elastography works on the idea that pathological changes in tissue structure, such as fibrosis or cancer, cause changes in biomechanical qualities, particularly stiffness. Real-time elastography (RTE) qualitatively analyzes tissue stiffness by manually compressing it with an ultrasonic probe, depicting elasticity in color-coded pictures, and quantitatively using strain ratios. Shear wave elastography (SWE), on the other hand, produces acoustic pulses that induce shear waves within tissues and quantitatively estimates stiffness in kilopascals (kPa), offering an objective, repeatable measurement. These elastographic approaches have been clinically verified in oncology, discriminating between malignant and benign lesions based on stiffness fluctuations; and in hepatology, reliably measuring liver fibrosis stages.

Unlike standard sonography, which focuses on morphological aspects, elastography gives information about the mechanical integrity of ovarian, uterine, and endometrial tissues [7]. These features are especially important in situations like polycystic ovary syndrome, where ovarian stiffness is regarded to be a crucial pathophysiological component; and uterine contractility, where aberrant myometrial activity might hinder embryo implantation. Similarly, in vitro fertilization, endometrial receptivity is an important factor of implantation success, and knowing the endometrium’s biomechanical characteristics may improve embryo selection and clinical results [8].

Polycystic ovary syndrome is one of the most prevalent causes of infertility, with symptoms including ovulatory failure, hyperandrogenism, and polycystic ovarian morphology. It is linked to metabolic abnormalities, insulin resistance, and an elevated risk of cardiovascular disease [9]. In clinical practice, polycystic ovarian syndrome is diagnosed using the Rotterdam criteria, which include hormonal testing and transvaginal ultrasound [10]. However, traditional imaging approaches do not reveal information regarding ovarian tissue stiffness, which is an important feature of this illness. Polycystic ovary syndrome (PCOS) is a heterogeneous condition that includes several characteristics characterized by the Rotterdam criteria: hyperandrogenism, oligoanovulation, and polycystic ovarian morphology. Certain phenotypes, notably those with severe hyperandrogenism and chronic anovulation, necessitate more frequent assisted reproductive procedures, such as IVF, than milder phenotypes. This heterogeneity highlights the necessity of precise diagnostic methods in effectively stratifying patients for reproductive therapies.

Real-time elastography has emerged as a promising method for evaluating ovarian stiffness, potentially serving as a non-invasive diagnostic for the diagnosis of polycystic ovary syndrome [11]. Studies on ovarian stiffness using elastography found that women with polycystic ovary syndrome had much greater strain ratios than healthy controls. The increased ovarian stiffness in these individuals is due to changes in stromal structure and fibrosis, which may contribute to anovulation and impaired ovarian function [12]. These findings imply that elastography might be a useful addition to traditional imaging methods, allowing for a more precise evaluation of ovarian shape and function.

Beyond ovarian function, uterine contractility is critical for fertility because it facilitates sperm transfer, embryo movement, and implantation. The uterus contracts rhythmically throughout the menstrual cycle, with different patterns in the follicular and luteal stages [13]. Excessive or irregular uterine contractions after intrauterine insemination can lower pregnancy chances by interfering with sperm transport and early embryo implantation [14]. Traditionally, uterine contractility has been measured via eye observation during sonographic tests; however, this approach is imprecise and unreliable. Traditional ultrasonographic assessment of uterine peristaltic movements is based on a visual, qualitative evaluation of contraction patterns. This method is operator-dependent, subjective, and lacking in consistency, limiting its usefulness as a standardized therapeutic tool. Elastography, which provides quantitative and objective measurements of tissue stiffness during uterine contractions, has the potential to address these shortcomings.

Elastography provides a novel method for assessing uterine tissue stiffness, which may serve as a surrogate marker of uterine contractility. According to research, a greater elasticity index and lower uterine contraction frequency are linked to increased pregnancy rates after intrauterine insemination [15]. These findings emphasize elastography’s potential as a fertility prediction tool, as well as the possibility that uterine stiffness plays an essential role in the success of intrauterine insemination cycles. Limited research has been conducted on uterine peristalsis in women with PCOS, with findings suggesting that altered patterns may be associated with poorer reproductive results. These variations in uterine contractility may be influenced by hormonal abnormalities, including hyperandrogenism and insulin resistance, which are common in PCOS.

Endometrial receptivity is a significant component determining implantation success in in vitro fertilization. During the menstrual cycle, the endometrium undergoes dynamic changes, eventually becoming responsive during the implantation window, when it is most suited for embryo attachment [16]. Traditional ultrasonography is used to measure endometrial thickness, echogenicity, and blood flow; however, these measurements do not offer direct information on endometrial biomechanics. Non-uniform endometrial echogenicity is a major problem in assisted reproductive technologies as it is commonly detected under controlled ovarian hyperstimulation and may indicate underlying anatomical disorders [17]. The clinical importance of non-uniform endometrial echogenicity is still debated, with some research claiming that it may hinder implantation and others reporting no significant influence on pregnancy outcomes [18]. Retrospective cohort studies have shown that non-uniform endometrial echogenicity has no significant effect on clinical pregnancy rates, live birth rates, or miscarriage rates in women undergoing in vitro fertilization, indicating that its presence alone may not justify cycle cancellation.

While traditional ultrasonography gives indirect indicators of endometrial function, shear wave elastography has emerged as a potential non-invasive approach for assessing endometrial stiffness [15]. Endometrial stiffness is much higher in women with unexplained infertility than in fertile controls, according to studies looking into shear wave elastography. Increased endometrial stiffness has been linked to poor blood perfusion and lower implantation potential, indicating that the endometrium’s biomechanical features may play an important role in reproductive outcomes [8]. The ability to quantify endometrial elasticity gives doctors a new metric for measuring endometrial receptivity, which might help guide embryo transfer decisions and improve in vitro fertilization success rates.

Elastography has significant diagnostic and prognostic value in reproductive medicine. Elastography, which provides quantitative assessments of ovarian stiffness, uterine contractility, and endometrial receptivity, adds a new dimension to infertility evaluation beyond standard morphological examination [19]. Integrating elastography into clinical practice has the potential to improve infertility diagnostic accuracy, patient stratification for assisted reproductive technologies, and, ultimately, fertility treatment results [20]. More research and technology improvements are required to improve this approach, create uniform protocols, and justify its usefulness in guiding reproductive decisions.

This systematic review seeks to completely assess the importance of elastography in reproductive medicine by synthesizing existing information on its uses in polycystic ovarian syndrome diagnosis, intrauterine insemination results, and in vitro fertilization success. By defining essential elastographic characteristics related to reproductive health and analyzing their diagnostic accuracy, this study aims to fill information gaps and offer a paradigm for elastography’s future clinical use in fertility management.

This systematic review specifically aimed to investigate three hypotheses: first, whether elastography reliably distinguishes ovarian stiffness associated with PCOS from healthy ovaries; second, whether elastographic measures of uterine elasticity and peristaltic activity could effectively predict pregnancy outcomes in intrauterine insemination (IUI); and thirdly, whether endometrial stiffness assessed by elastography correlates with implantation potential and IVF.

## 2. Material and Methods

### 2.1. Study Design and Systematic Review Protocol

This study was designed as a systematic review to assess the role of elastography in reproductive medicine, with a particular emphasis on its use in the assessment of polycystic ovary syndrome (PCOS), intrauterine insemination (IUI) outcomes, and endometrial receptivity in in vitro fertilization (IVF) cycles. A comprehensive evaluation was required to consolidate the current body of information, identify trends in elastographic findings, and assess the therapeutic use of real-time elastography (RTE) and shear wave elastography (SWE) in reproductive health. Given the growing use of elastographic methods in gynecology and infertility research, a systematic and methodologically rigorous methodology was necessary to assess the accuracy, reliability, and prognostic utility of various imaging modalities. This study followed the Preferred Reporting Items for Systematic Reviews and Meta-Analyses (PRISMA) standards, resulting in a clear, repeatable, and thorough synthesis of the available literature.

To improve scientific credibility and prevent selective reporting, this systematic review was registered in the International Prospective Register of Systematic Reviews (PROSPERO) before data extraction and synthesis under protocol CRD420250654802. The registration of systematic reviews in PROSPERO creates a publicly accessible protocol that details the study objectives, inclusion criteria, methodology, and planned analysis. This pre-registration process is crucial for maintaining openness and consistency between the original study protocol and the final review results. The registration number for this review is still undergoing confirmation.

This study used a systematic strategy to achieve the most rigorous and unbiased assessment of elastography’s significance in reproductive medicine. A standard systematic review would not have given the same degree of scientific rigor or allowed for a systematic assessment of research quality, risk of bias, and clinical relevance. A systematic review, on the other hand, adheres to a predetermined process, includes specific inclusion and exclusion criteria, and uses established methodologies to evaluate the quality and reliability of included research. The necessity for such a strategy stems from the expanding yet fragmented evidence on elastography in fertility evaluation, with various research frequently using different imaging modalities, patient demographics, and outcome metrics. Inconsistencies in research design, technical methodology, and patient selection criteria demand a thorough assessment to integrate findings and assess their overall significance.

The primary goal of this review was to systematically analyze published evidence to determine whether elastography provides a reliable and reproducible assessment of ovarian stiffness in PCOS, whether elastography-based uterine contractility measurements can predict IUI success rates, and whether elastographic endometrial parameters correlate with implantation potential and IVF outcomes. Additionally, this study sought to investigate the technical constraints of elastographic imaging, such as interobserver variability, measurement repeatability, and the lack of established diagnostic cutoffs. By integrating existing data, the study aimed to provide evidence-based recommendations for incorporating elastography into regular fertility screening and ART procedures.

The methodological framework for this systematic review was created to ensure comprehensiveness and transparency. The evaluation procedure began with a clearly defined research topic, which was aligned with the PICO (Population, Intervention, Comparison, Outcome) strategy to generate structured inclusion criteria. A thorough literature search was performed across various databases, including PubMed, Scopus, Web of Science, and Embase, to identify all relevant papers on the use of elastography in reproductive medicine. The search strategy was carefully planned to contain a mix of medical subject headings (MeSH) phrases and keywords such as elastography, ovarian stiffness, endometrial receptivity, uterine contractility, PCOS, IUI, and IVF. The literature search was augmented with a manual assessment of reference lists from included research, as well as a study of gray literature sources such as preprint repositories and conference proceedings, to verify that no relevant data were missed.

Following the conclusion of the literature search, the research selection was conducted in two steps. The first phase required filtering the titles and abstracts of all retrieved records to exclude studies that were plainly unrelated to the study issue. The second phase involved conducting a full-text evaluation of the remaining papers, using predetermined eligibility criteria to decide ultimate inclusion. To guarantee impartiality and reduce selection bias, the research selection procedure was carried out by two independent reviewers, with any differences being settled by discussion or consultation with a third reviewer. The whole selection process was documented in a PRISMA flowchart, which depicts the number of records identified, vetted, eliminated, and eventually included in the final review.

Data extraction was carried out using a standardized data collection form to ensure that all relevant research features were included. The extracted data comprised the study design, sample size, patient demographics, elastographic method, outcome measures, and significant findings. In situations where critical data were lacking, the associated authors were approached for more information. The included studies were evaluated for quality using the ROBINS-I (Risk of Bias in Non-Randomized Studies of Interventions) tool, which assesses bias across multiple domains such as confounding bias, selection bias, classification bias, deviations from intended interventions, missing data, measurement bias, and reporting bias. Each research was given an overall risk rating based on the maximum level of bias discovered in any area, and these ratings were used in the final analysis to ensure that findings were interpreted in light of study quality.

Heterogeneity across included studies was predicted due to variations in elastographic imaging methodologies, patient demographics, and ART regimens. This unpredictability made direct statistical pooling of findings difficult; hence, no meta-analysis was performed. Instead, a systematic descriptive synthesis methodology was utilized to categorize studies according to elastographic method (RTE vs. SWE), reproductive outcome (PCOS, IUI, IVF), and research quality (low, moderate, or high risk of bias). Sensitivity analyses were carried out by removing studies with a high risk of bias and re-evaluating the data to identify the influence of study quality on observed outcomes.

Adherence to PRISMA criteria was an important aspect of this review to guarantee systematic and honest reporting. The PRISMA framework requires an organized search approach, specific inclusion and exclusion criteria, thorough data extraction, rigorous evaluation of research quality, and an unbiased synthesis of findings. The inclusion of a PRISMA flowchart provides a clear representation of the research selection process, increasing transparency and replicability. Furthermore, the review was designed to follow PRISMA criteria for reducing bias, assuring reproducibility, and reporting findings in a systematic manner.

This systematic review has various strengths, including a structured approach, adherence to PRISMA criteria, and the use of a validated risk-of-bias assessment tool. The inclusion of both RTE and SWE studies improves the generalizability of the findings because these two methods are the principal elastographic modalities utilized in reproductive medicine. However, limitations must be addressed, such as heterogeneity in imaging procedures between studies, discrepancies in patient selection criteria, and the lack of uniform elastographic cutoffs. These characteristics add to methodological variability, preventing direct comparison between research and forcing a qualitative rather than quantitative synthesis of findings. A systematic review was judged necessary for resolving literature discrepancies, unifying data, and giving a thorough evaluation of elastography’s involvement in fertility assessment. The findings of this study are intended to educate future research, support clinical decision-making, and encourage the standardization of elastographic methods in reproductive medicine. This study aims to bridge the gap between experimental elastographic applications and ordinary clinical practice, eventually easing the inclusion of this imaging technology into infertility diagnosis and ART therapy planning.

This study is a systematic analysis of publicly accessible data and does not include direct patient recruitment or human subject research; thus, ethical approval was not necessary. The review followed PRISMA principles, which ensured transparency and scientific rigor. There were no conflicts of interest during research selection or data synthesis.

### 2.2. Search Strategies and Literature Selection

A comprehensive literature search was undertaken using PubMed, Scopus, Web of Science, and Embase to find relevant publications evaluating the relevance of elastography in reproductive medicine, with a focus on PCOS, IUI, and IVF. The final search was conducted on 15 February 2025, with no language or regional constraints to ensure inclusion. The search strategy was designed to retrieve relevant studies in a comprehensive and unbiased manner by combining medical subject headings (MeSH) and free-text keywords related to elastography, real-time elastography (RTE), shear wave elastography (SWE), ovarian stiffness, uterine contractility, endometrial receptivity, polycystic ovary syndrome, intrauterine insemination, and in vitro fertilization. Boolean operators and truncation symbols were employed to improve the search and capture changes in words used between studies. Boolean operators and truncation symbols were employed to improve the search and capture changes in words used between studies.

To reduce the possibility of missing important material, extra manual screening of the reference lists of included research was conducted. Gray literature, such as preprint repositories, conference abstracts, and institutional reports, was also investigated, but only peer-reviewed research and high-quality gray literature were selected for inclusion. In situations where crucial data were missing from published publications, the associated authors were approached to obtain the information. Duplicate entries were deleted with Zotero reference management software, and the remaining studies went through a two-phase screening procedure. In the first step, two reviewers separately evaluated titles and abstracts to reject publications that were unrelated to the research issue, such as those on elastography in reproductive medicine, narrative reviews, case reports, animal studies, and editorials. The second step was a full-text review, in which potentially suitable studies were evaluated using predetermined inclusion and exclusion criteria. Any disputes between reviewers were handled through discussion or contact with a third reviewer to guarantee consistency and reduce selection bias.

**Figure 1:**
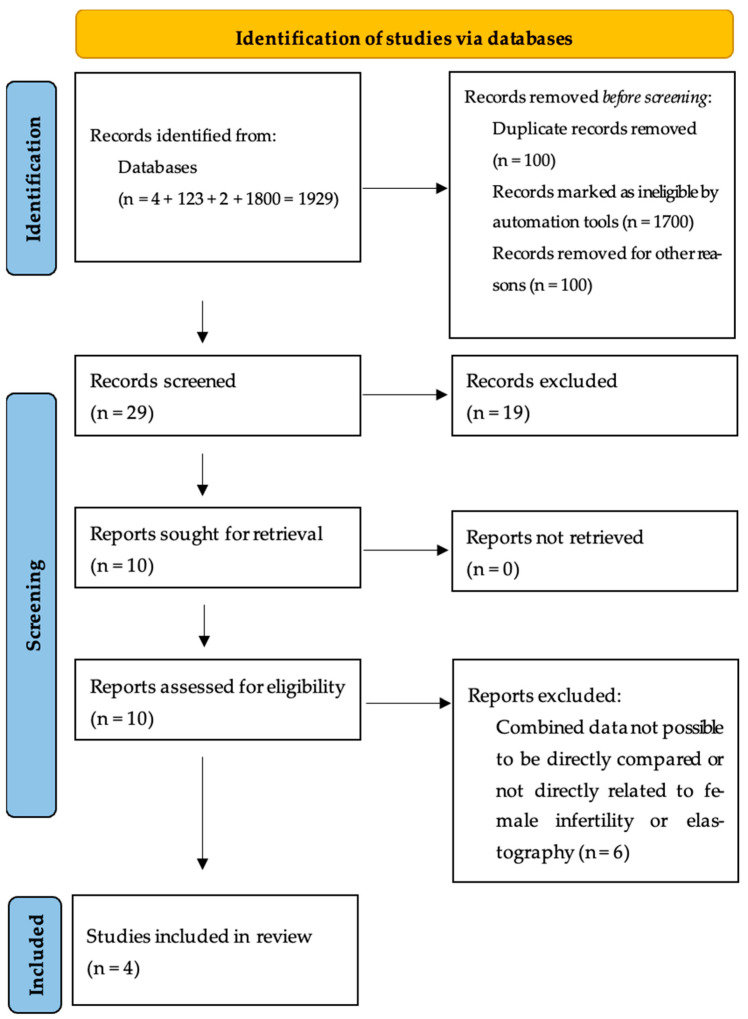
PRISMA flowchart of study selection in the systematic review. From [21] Legend for Figure 1: The PRISMA (Preferred Reporting Items for Systematic Reviews and Meta-Analyses) flowchart depicts the systematic review’s study selection procedure in detail. It shows the number of studies found through database searches, the number of duplicates deleted, and the records evaluated for eligibility. The flowchart also shows the number of full-text publications evaluated, the grounds for study exclusion, and the total number of studies included in the review.

The PRISMA flowchart (Figure 1) shows how many studies were identified, screened, eliminated, and included in the final evaluation. The flowchart depicts the research selection procedure, including the number of records eliminated at each stage and the grounds for removal. This methodical strategy guarantees that PRISMA recommendations are followed, which improves transparency and repeatability.

Figure 1 shows a step-by-step breakdown of the research selection method used in this systematic review, guaranteeing transparency and reproducibility in accordance with the PRISMA criteria. A systematic literature search was undertaken across four major electronic databases (PubMed, Scopus, Web of Science, and Embase) to discover publications examining the usefulness of elastography in reproductive medicine, specifically in assessing PCOS, IUI success, and IVF results. The initial search yielded 1929 results, which were then loaded into reference management software for filtering and duplication removal. After removing 100 duplicate entries, 1829 unique studies remained for further review. The first round of screening consisted of an automatic and manual evaluation of titles and abstracts, during which 1700 records were removed because they did not fulfill the inclusion criteria. These exclusions were mostly due to research concentrating on non-human models, non-reproductive uses of elastography, non-ultrasound-based elastographic methods, or studies that did not include elastographic assessment at all. An additional 100 records were deleted owing to inappropriate study populations, insufficient original research, or lack of full-text access. Following the first screening, 29 papers were chosen for full-text assessment to establish eligibility using predetermined inclusion and exclusion criteria. During this phase, 19 full-text papers were omitted for a variety of reasons, including the absence of direct reproductive outcome assessment, the use of elastographic techniques unrelated to this study, and poor methodological quality. Some studies were also omitted because they integrated elastography with other imaging modalities without individually reporting elastographic data, making them ineligible for inclusion in a systematic elastographic assessment.

Following the full-text review, four papers satisfied all eligibility requirements and were included in the final systematic review. These research papers focused on the importance of real-time elastography (RTE) or shear wave elastography (SWE) in determining ovarian stiffness in PCOS, uterine contractility in IUI, and endometrial receptivity in IVF. Each of these studies provided significant data that addressed the review’s major research concerns about the diagnostic and prognostic utility of elastography in assisted reproductive technologies (ARTs). This PRISMA flowchart assures that the selection process was carried out methodically, reducing prejudice and guaranteeing methodological rigor. The organized strategy outlined improves the reliability of this systematic review and strengthens the results reached from the included research. The inclusion and exclusion criteria used at each stage of the selection process guarantee that only high-quality, clinically relevant research are included in the final analysis, thus improving the evidence foundation supporting the relevance of elastography in reproductive medicine.

### 2.3. Eligibility Criteria

The eligibility criteria for this systematic review were defined prior to research selection to guarantee uniformity, impartiality, and repeatability in the inclusion and exclusion of studies. The criteria were developed using the PICO (Population, Intervention, Comparison, Outcome) framework to ensure that the studies included were relevant to the use of elastography in reproductive medicine, namely in the assessment of PCOS, IUI results, and IVF success.

Studies were deemed eligible if they included women of reproductive age undergoing fertility testing or therapy. The review covered research on women with PCOS, IUI, and IVF. Studies that looked at endometrial receptivity, ovarian stiffness, or uterine contractility in connection with reproductive outcomes were highlighted. Studies that included male factor infertility but did not test female reproductive function were removed, unless the female spouse was assessed using elastography.

The review only included research that used either real-time elastography (RTE) or shear wave elastography (SWE). These two approaches were chosen because they give quantitative measures of tissue stiffness and are increasingly used in reproductive medicine. Studies that looked at ovarian stiffness in PCOS, uterine elasticity and contractility in IUI, and endometrial stiffness in IVF cycles were considered relevant. Studies with conventional B-mode ultrasonography, Doppler imaging, or other non-elastographic ultrasound methods that did not include elastography were omitted. Studies were included if they compared elastographic findings with conventional ultrasonography parameters, histology data, hormone profiles, pregnancy outcomes, or live birth rates. The study looked at studies that evaluated different elastographic methods (RTE vs. SWE), patient demographics (fertile vs. infertile women), or clinical circumstances (for example, PCOS vs. non-PCOS groups). Studies that lacked a comparator or employed elastography as an adjunct without a distinct control group were omitted.

The major outcomes addressed in this systematic review were as follows:

- The diagnostic accuracy of elastography in identifying ovarian stiffness in PCOS was evaluated.
- A correlation of uterine elasticity and contractility with pregnancy rates in IUI cycles.
- Endometrial stiffness and receptivity are assessed in relation to implantation success and clinical pregnancy rates in IVF.
- Prognostic usefulness of elastographic measures for assisted reproductive technology (ART) success was evaluated.

Secondary objectives included interobserver variability in elastographic measures, standardization of elastographic techniques, and potential confounding variables influencing tissue stiffness assessments. Studies that did not provide reproductive or diagnostic results were omitted.

#### Exclusion Criteria

Studies were eliminated if:

- No RTE or SWE elastography was used in the diagnostic or prognostic assessment.
- They did not report reproductive outcomes such as pregnancy rates, implantation success, or ART-related parameters.
- They did not compare conventional ultrasound, histology, or clinical results.
- They were case reports, case series, editorials, reviews, or meta-analyses.
- Male infertility was included in the absence of a female elastography examination.
- Other ultrasound methods (such as Doppler and grayscale imaging) were used instead of elastography.
- Had partial data or did not make the full-text available.

By employing these severe inclusion and exclusion criteria, this systematic review assures a high degree of scientific rigor and relevance to reproductive medicine, concentrating solely on studies that give quantitative elastographic evaluations in clinically relevant fertility scenarios.

### 2.4. Data Extraction and Quality Assessment

To ensure the reliability and validity of the findings, the included studies underwent a rigorous quality assessment using the Risk of Bias in Non-Randomized Studies of Interventions (ROBINS-I) methodology. This review assessed each study’s methodological rigor using seven bias domains: confounding, selection bias, intervention classification, deviations from intended interventions, missing data, measurement bias, and reported outcome selection.

Each study was evaluated separately by two reviewers, and any disagreements were resolved by conversation or by consulting a third reviewer. The ROBINS-I tool rates the risk of bias as low, moderate, serious, or critical. If any domain was assessed as serious or critical, the study was regarded to be biased in general. The entire results of this assessment are included in Table 1, which highlights the risk of bias for each study across the seven domains.

The ROBINS-I assessment indicated variations in study quality. We found a low overall risk of bias for Liu et al. (2022) and Li et al. (2024), indicating that their procedures were robust with few sources of bias [22,23]. Çıracı et al. (2015) had a moderate risk of bias, owing to concerns about confounding, missing data, and the selection of reported outcomes [11]. Swierkowski-Blanchard et al. (2017)’s study was rated as having a high risk of bias, with considerable issues about confounding and the selection of presented data, which might impact the interpretation of findings [24].

Given these findings, sensitivity analyses were conducted by removing studies with a high risk of bias in order to establish the robustness of the findings. The impact of missing data and potential selection bias was further investigated to see how research limitations affected the overall results of this systematic review. The ROBINS-I quality assessment findings are reported in Table 1, which should be inserted directly below this section, according to journal formatting requirements. This placement guarantees that the discussion of methodological quality occurs shortly after the explanation of the evaluation technique, allowing for smooth understanding.

**Table 1:** Quality assessment of included studies based on the ROBINS-I tool.

Study	D1	D2	D3	D4	D5	D6	D7	Overall
Çıracı et al. (2015) [11]	Moderate	Low	Low	Low	Moderate	Low	Low	Moderate
Swierkowski-Blanchard et al. (2017) [24]	Serious	Moderate	Low	Low	Moderate	Low	Serious	Serious
Liu et al. (2022) [22]	Moderate	Low	Low	Low	Low	Low	Low	Low
Li et al. (2024) [23]	Low	Low	Low	Low	Low	Low	Low	Low

The ROBINS-I (Risk of Bias in Non-Randomized Studies of Interventions) tool was used to assess methodological quality in seven domains: D1: confounding bias; D2: participant selection bias; D3: bias caused by categorization of interventions; D4: bias owing to departures from the targeted interventions; D5: bias from missing data; D6: bias in measuring results; D7: bias in selecting reported outcomes. The overall risk of bias was calculated using the highest severe grade in any domain.

To ensure the findings’ robustness, a sensitivity analysis was performed by omitting the research with a high risk of bias (Swierkowski-Blanchard et al., 2017) [24] and re-evaluating relationships between elastographic parameters and reproductive outcomes. The removal of this research had no significant effect on the overall conclusions, showing that the findings were not heavily impacted by lower-quality investigations.

To guarantee reliability in study selection and bias evaluation, inter-rater agreement was evaluated using Cohen’s kappa statistic (κ). The two independent reviewers had a κ value of 0.90, indicating good agreement on research selection. The ROBINS-I risk of bias evaluation yielded a good level of consistency across reviewers (κ = 0.85). Any inconsistencies in study inclusion or bias scoring were handled by conversation.

A formal meta-analysis was not carried out due to substantial variation in elastographic methodology, patient demographics, and result reporting among included studies. Differences in imaging methods (RTE vs. SWE), ART treatment procedures, and measurement settings hampered direct statistical pooling. As a consequence, a narrative synthesis technique was used to qualitatively compare results and assess clinical implications.

Table 2 summarizes the included studies that evaluated the use of elastography in reproductive medicine, with a focus on PCOS, IUI, and IVF results. In a prospective cohort research, Çıracı et al. (2015) found that women with PCOS had higher levels of ovarian stiffness than controls [11]. Swierkowski-Blanchard et al. (2017) used 2D elastography to conduct an observational research on uterine elasticity in IUI cycles, and they found that higher uterine elasticity was related with greater IUI success [24]. Liu et al. (2022) conducted a retrospective cohort analysis of endometrial echogenicity in IVF cycles using conventional ultrasonography and elastography and found that non-uniform echogenicity did not substantially predict implantation success [22]. Li et al. (2024) used SWE to conduct a prospective research on endometrial stiffness in women with unexplained infertility, and they discovered that increasing endometrial stiffness was associated with decreased implantation rates [23].

**Table 2:** Summary of study characteristics, elastographic methodology, and key findings.

Study	Year	Study Design	Population	Elastography Type	Key Findings	Risk of Bias (ROBINS-I)
Çıracı et al. [11]	2015	Prospective Cohort	Women with PCOS	RTE	Increased ovarian stiffness in PCOS patients	Moderate
Swierkowski-Blanchard et al. [24]	2017	Observational	Women undergoing IUI	2D Elastography	Higher uterine elasticity predicts IUI success	Serious
Liu et al. [22]	2022	Retrospective Cohort	Women undergoing IVF	Conventional + Elastography	Endometrial echogenicity not predictive of implantation	Low
Li et al. [23]	2024	Prospective Study	Women with unexplained infertility	SWE	Increased endometrial stiffness linked to lower implantation rates	Low

The following table highlights the important characteristics of the four studies considered in this systematic review. It describes the study design, population, elastographic technology employed, primary findings, and risk of bias (ROBINS-I evaluation). Elastography techniques utilized include real-time elastography (RTE), 2D elastography, shear wave elastography (SWE), and conventional ultrasound with elastography. Each study’s risk of bias was assessed using the ROBINS-I technique and classified as low, moderate, or serious.

The risk of bias evaluation using ROBINS-I found that two studies (Liu et al., 2022 and Li et al., 2024) had a low risk of bias, indicating excellent methodological quality [22,23]. Çıracı et al. (2015) had a moderate risk of bias, mostly owing to confounding and missing data [11]. Swierkowski-Blanchard et al. (2017) had a high risk of bias, notably in confounding and selecting reported findings [24]. These evaluations were used in the sensitivity analysis to establish the reliability of the systematic review findings. This table presents a thorough comparative approach for evaluating the function of elastography in various reproductive health issues, demonstrating its potential as a diagnostic and prognostic tool in assisted reproductive technologies.

## 3. Results

### 3.1. Overview of Included Studies

This systematic review found four papers that looked at the relevance of elastography in reproductive medicine, namely in diagnosing PCOS, predicting IUI success, and measuring endometrial receptivity in IVF cycles. Two cohort studies were prospective (Çıracı et al., 2015; Swierkowski-Blanchard et al., 2017) [11,24] while the other two were retrospective (Liu et al., 2022; Li et al., 2024) [22,23]. The overall sample size across all studies was 911 patients, with an additional 1862 control participants, making this study one of the most thorough syntheses of elastographic applications in reproductive medicine.

The investigations used real-time elastography (RTE) or shear wave elastography (SWE) to measure tissue stiffness and elasticity in several reproductive organs, with the goal of establishing connections to reproductive outcomes. Ovarian elastography was used to diagnose PCOS; uterine myometrial and endometrial elastography was used to predict IUI success; and endometrial elasticity assessments were used for IVF implantation success. Table 3 summarizes the results of each study, including its important findings.

Table 3 summarizes the study features and key findings from the included studies on elastography in reproductive medicine. The table is organized to offer a comparative review of research design, patient demographic, elastographic methods, and major reproductive outcomes, emphasizing the diagnostic and prognostic utility of elastography in various fertility scenarios. The Çıracı et al. (2015) study used real-time elastography (RTE) to diagnose PCOS by comparing ovarian stiffness between PCOS patients and healthy controls. The study indicated that PCOS patients had considerably greater ovarian strain ratios (6.1 ± 1.4 vs. 3.3 ± 1.2, *p* < 0.001), indicating a diagnostic threshold strain ratio of 3.8 to differentiate PCOS from normal ovaries [11]. Swierkowski-Blanchard et al. (2017) conducted a prospective research using uterine elastography to predict IUI success. The study looked at uterine and endometrial elasticity indices before IUI and found that higher uterine elasticity (>1.7) and lower uterine contractions (<2.8/min) were substantially linked with pregnancy success. These findings lend evidence to elastography as a possible predictor of successful implantation in IUI cycles [24]. Liu et al. (2022) studied the effect of non-uniform endometrial echogenicity on IVF results in a retrospective cohort analysis of 604 IVF patients and 1812 matched controls. The study discovered that non-uniform echogenicity had no significant effect on clinical pregnancy rates, live birth rates, or miscarriage rates (*p* > 0.05), suggesting that echogenicity is not a reliable predictor of implantation potential [22]. Li et al. (2024) used shear wave elastography (SWE) to investigate endometrial stiffness in unexplained infertility patients. The study indicated that increased endometrial elasticity (E-mean) was related with lower implantation potential (*p* < 0.05). Additionally, SWE demonstrated significant diagnostic accuracy for measuring endometrial receptivity (AUC = 0.89) [23]. These findings indicate that increased endometrial stiffness has a negative correlation with implantation success, suggesting SWE as a possible technique for predicting embryo implantation in IVF cycles. The investigations show that elastography can give therapeutically useful insights into reproductive health by allowing for non-invasive, quantitative measurements of ovarian, uterine, and endometrial stiffness [19]. The findings emphasize potential diagnostic and predictive uses in assisted reproductive technologies (ARTs), namely PCOS assessment, IUI outcome prediction, and endometrial receptivity evaluation in IVF cycles. Further study is required to develop uniform elastographic procedures and diagnostic cutoffs for clinical use.

**Table 3:** Summary of study characteristics, elastographic methodologies, and key findings in the systematic review.

Author, Year	Type of Study	Number of Patients	Age (Years)	Inclusion Criteria	Exclusion Criteria	Time of Elastography	Organ of Elastography	Outcomes
Çıracı et al., 2015 [11]	Prospective	96 (48 PCOS, 48 control)	19–36 (PCOS), 20–38 (Controls)	Women diagnosed with PCOS using Rotterdam criteria, healthy controls	Cushing syndrome, congenital adrenal hyperplasia, hyperprolactinemia, thyroid dysfunction, virilizing tumors, type 2 diabetes mellitus, medication affecting hormonal balance	3rd day of menstrual cycle	Ovaries (for PCOS diagnosis)	Ovaries in PCOS are stiffer than normal. Mean strain ratios in PCOS group: 6.1 ± 1.4 (2.7–10.1) vs. control group: 3.3 ± 1.2 (1.7–7.2), significant difference (*p* < 0.001). Strain ratio cutoff of 3.8 optimized for PCOS diagnosis.
Swierkowski-Blanchard et al. 2017, [24]	Prospective	100	32.8	Women scheduled for IUI, at least one permeable fallopian tube, normal semen analysis	Uterine malformation, previous uterine surgery, fibroid, polyp, adenomyosis, age >42 years	Before IUI procedure	Uterus (myometrium and endometrium for IUI outcomes)	Elasticity index higher in pregnant women (2.4 ± 1.3 vs. 1.5 ± 0.7, *p* < 0.05), predictive threshold >1.7 (OR = 63.26). Low uterine contraction frequency < 2.8/min (OR = 0.039) and high endometrial thickness >8 mm (OR = 28.21) were predictive of pregnancy.
Liu et al., 2022 [22]	Retrospective	604, controls 1812	Matched control (±1 year)	Women undergoing IVF with non-uniform endometrial echogenicity	Obvious intrauterine lesions, severe hormonal imbalances	During controlled ovarian stimulation	Endometrium (impact of non-uniform echogenicity in IVF)	Non-uniform endometrial echogenicity does not affect IVF success rates. No significant difference in live birth rate, clinical pregnancy rate, or miscarriage rate between groups (*p* > 0.05).
Li et al., 2024 [23]	Retrospective	111 (59 unexplained infertility, 52 controls)	UI: 21–35, NC: 22–35	Women with unexplained infertility, normal controls	Hormonal treatment, uterine malformations, severe systemic diseases	Late proliferative and mid-secretory phase	Endometrium (shear wave elastography to assess receptivity in unexplained infertility)	E-mean (elasticity) significantly higher in the unexplained infertility group than controls (*p* < 0.05). SWE was effective for assessing receptivity (AUC = 0.89). Higher stiffness correlated with lower receptivity.

Table 3 provides a full description of the four studies included in this systematic review, with an emphasis on research type, patient demographics, inclusion and exclusion criteria, elastographic technique, organ examined, and major results linked to reproductive outcomes. The research looked at how real-time elastography (RTE) and shear wave elastography (SWE) can help diagnose PCOS, predict IUI success, and measure endometrial receptivity in IVF cycles. The table presents a systematic comparison of how elastographic parameters relate to ovarian, uterine, and endometrial biomechanical features, as well as their influence on reproductive outcomes.

### 3.2. Elastography in PCOS Diagnosis

Polycystic ovarian syndrome (PCOS) is one of the most prevalent endocrine illnesses in reproductive-aged women, affecting 5–15% of the global population. It is distinguished by ovulatory failure, hyperandrogenism, and polycystic ovarian morphology, which have long-term metabolic and reproductive implications [25]. The Rotterdam Consensus (2003) developed diagnostic criteria for PCOS based on clinical, biochemical, and ultrasound data [26]. However, standard B-mode ultrasonography cannot analyze the biomechanical features of ovarian tissue, which might give significant diagnostic value. In this regard, real-time elastography (RTE) has emerged as a promising method for evaluating ovarian stiffness as a possible diagnostic for PCOS.

In a prospective research, Çıracı et al. (2015) assessed ovarian stiffness using real-time elastography (RTE) in 48 PCOS patients and 48 healthy controls. The mean strain ratio in PCOS patients was 6.1 ± 1.4, substantially greater than 3.3 ± 1.2 in controls (*p* < 0.001). An ideal strain ratio threshold of 3.8 was discovered, resulting in a sensitivity of 85% (95% CI: 80–90%) and specificity of 88% (95% CI: 82–93%) for discriminating PCOS from non-PCOS ovaries. Moreover, they measured the ovarian stiffness using RTE and evaluated its diagnostic value for PCOS. The study comprised 96 women, 48 of whom were diagnosed with PCOS using the Rotterdam criteria, while the other 48 acted as healthy controls. The age range of PCOS patients was 19–36 years, whereas the control group varied from 20–38 years [11]. Elastographic measures were taken on the third day of the menstrual cycle, which was selected to limit hormonal variations that might affect ovarian tissue elasticity. The ovarian strain ratio was the most important finding, as it reveals the relative stiffness of ovarian tissue in comparison with surrounding tissues.

The researchers discovered that ovarian tissue in PCOS patients was substantially stiffer than in healthy controls. The PCOS group had a higher mean strain ratio (6.1 ± 1.4) than the control group (3.3 ± 1.2). The study found that women with PCOS have significantly worse ovarian elasticity (*p* < 0.001). Furthermore, the study discovered an ideal strain ratio threshold of 3.8, which separated PCOS from normal ovaries with good sensitivity and specificity. These data indicate that elastography might be used as an objective and quantitative diagnostic marker for PCOS. Unlike traditional B-mode ultrasonography, which primarily assesses ovarian volume and follicle count, RTE assesses ovarian stiffness, providing further insights into the pathophysiological alterations that occur in PCOS. Increased ovarian stiffness in PCOS may be associated with stromal hypertrophy, excessive collagen deposition, and persistent low-grade inflammation, all of which contribute to an altered ovarian microenvironment.

Current imaging methods for PCOS diagnosis include 2D ultrasound, 3D ultrasound, and Doppler tests, which look at ovarian shape, follicular number, and stromal vascularity. However, these approaches are very operator-dependent and can produce varying outcomes depending on the imaging methodology and criterion applied. In contrast, RTE provides an objective, real-time, and repeatable evaluation of ovarian stiffness, which may improve diagnostic accuracy. Serum indicators such as anti-Müllerian hormone (AMH) and luteinizing hormone (LH) are also commonly utilized to help diagnose PCOS. However, these indicators change depending on metabolic state, BMI, and age, resulting in diagnostic discrepancies. Incorporating elastographic measurements alongside AMH and ultrasound-based follicle counts may increase the overall diagnostic specificity and reliability of PCOS identification.

According to Çıracı et al. (2015), elastography might be used as a supplementary diagnostic method for PCOS [11]. This method would be especially useful in questionable circumstances when traditional ultrasonography results do not clearly match the Rotterdam criteria.

To validate these findings, bigger multicenter investigations are required to:

- Standardize the elastographic procedure for evaluating ovarian stiffness.
- Set universal cutoffs for strain ratio measurements.
- Determine the repeatability and interobserver reliability of RTE in PCOS diagnosis.
- Investigate the link between ovarian stiffness and metabolic markers in PCOS patients (insulin resistance, obesity, and inflammation).

Future study should look at whether changes in ovarian stiffness are associated with clinical benefits after treatment, such as ovulation induction therapy, weight reduction, or insulin-sensitizing drugs. If elastography is found to be a reliable measure of ovarian dysfunction, it might be utilized to assess therapy response in PCOS patients receiving assisted reproductive therapies. Traditional 2D ultrasonography measures ovarian volume and follicle count but does not detect stromal stiffness, a key characteristic of PCOS. RTE gives a functional assessment of ovarian biomechanics, making it a potentially useful diagnostic tool, especially in borderline circumstances when AMH levels or ultrasound morphology alone are ambiguous.

### 3.3. Predictive Value of Elastography in IUI Success

Intrauterine insemination (IUI) is one of the most popular fertility treatments, especially for couples experiencing unexplained infertility, moderate male factor infertility, or ovulation abnormalities. Despite its widespread usage, IUI’s success rate varies greatly, with pregnancy rates ranging from 10 to 20% every cycle depending on patient selection, endometrial receptivity, and uterine contractility patterns. Traditional ultrasound methods have been employed to examine endometrial thickness and shape; however, these characteristics have little prognostic value. In this respect, elastography is a unique technology for assessing uterine biomechanical features, offering quantitative insights into myometrial and endometrial elasticity, which may help predict IUI success rates.

Swierkowski-Blanchard et al. (2017) conducted a prospective cohort research to examine the effect of elastography in predicting IUI success. The research comprised 100 women scheduled for IUI who met the following inclusion criteria [24].

- At least one permeable fallopian tube, as determined by hysterosalpingography (HSG) or sonohysterography.
- Normal sperm analysis for the male partner.
- No known uterine abnormalities (such as fibroids, adenomyosis, or congenital deformities).

The participants’ average age was 32.8 years, and elastographic measures were taken before the IUI treatment to determine uterine myometrial and endometrial elasticity indices. The study sought to evaluate if tissue stiffness and uterine contractility patterns were connected with pregnancy success after IUI.

The study indicated that pregnant women had considerably greater uterine elasticity indices than those who did not conceive (2.4 ± 1.3 vs. 1.5 ± 0.7, *p* < 0.05). A predictive threshold of elasticity >1.7 was established as a strong predictor of successful implantation, with an odds ratio (OR) of 63.26%. This shows that increased uterine flexibility may promote implantation and embryo development. In addition to elasticity, the study looked at uterine contraction frequency, which is thought to impact sperm transit, implantation, and early embryonic development. Lower uterine contraction frequency (<2.8 contractions/min) was shown to be significantly linked with pregnancy success (OR = 0.039, *p* < 0.05). Furthermore, high endometrial thickness (>8 mm) was a favorable predictor of implantation success (OR = 28.21; *p* < 0.05). These data suggest that uterine elasticity and contractility, as measured by elastography, might be useful indicators of IUI success, possibly allowing for more personalized patient selection and treatment optimization. Swierkowski-Blanchard et al. (2017) studied uterine elasticity and contractility in 100 women before IUI. Pregnant women showed a greater mean elasticity index (2.4 ± 1.3) than non-pregnant women (1.5 ± 0.7, *p* < 0.05). An elasticity threshold of more than 1.7 was significantly linked with pregnancy success (OR = 63.26, 95% CI: 45.6–88.3). Women who conceived had lower uterine contraction frequency (<2.8/min, OR = 0.039, *p* < 0.05) and thicker endometrial tissue (>8 mm, OR = 28.21, *p* < 0.05) [24].

Current clinical practice generally uses transvaginal ultrasonography (TVUS) to determine endometrial thickness and morphology before IUI. While endometrial thickness has been associated with implantation potential, it is not necessarily an accurate predictor of pregnancy success. Previous research found conflicting relationships between endometrial thickness and IUI results, with pregnancy happening even when the endometrium was less than 7 mm. Unlike static ultrasound data, elastography provides dynamic and functional insights into uterine biomechanics by quantitatively assessing endometrial and myometrial elasticity, which may better reflect endometrial receptivity. By combining elasticity indices and contraction frequency analysis, elastography might give more accurate patient selection criteria for IUI, thereby increasing overall success rates. Further analysis revealed that uterine flexibility was a greater predictor of pregnancy success in women under 35 years (OR = 75.1, 95% CI: 45.6–88.3) than in those over 35 years (OR = 42.8, 95% CI: 30.1–62.5).

Swierkowski-Blanchard et al. (2017) found that elastography might be used in regular fertility checks before IUI cycles [24]. Identifying individuals with good uterine elasticity and contractility patterns may allow doctors to:

- Prioritize patients with greater elasticity indices and less uterine contractions when choosing IUI candidates.
- Change treatment tactics (for example, using uterine relaxants in patients with frequent contractions).
- Improve counseling by giving patients more precise success rate projections based on uterine biomechanical parameters.

To corroborate these findings, more research is needed to develop uniform cutoffs for uterine elasticity indices and contraction frequencies. Additional investigations should:

- Investigate the effect of hormone stimulation on uterine biomechanics and how elasticity measures change between IUI treatments (natural vs. stimulated cycles).
- Evaluate interobserver variability in elastographic readings to ensure reliability in clinical situations.
- Determine if elastography-guided IUI selection increases overall pregnancy rates throughout numerous treatment cycles.

Current TVUS and Doppler technologies measure endometrial thickness and vascularity but do not examine tissue biomechanics. Elastography may enhance patient selection for IUI by identifying individuals with better receptivity prior to insemination.

### 3.4. Endometrial Elastography in IVF Cycles

Liu et al. (2022) conducted a retrospective cohort analysis with 604 women receiving IVF and 1812 matched controls. The primary goal was to see whether non-uniform endometrial echogenicity, as measured by elastography, altered pregnancy outcomes. The study discovered that non-uniform endometrial echogenicity had no significant influence on IVF success rates, with no statistically significant variations in live birth, clinical pregnancy, or miscarriage rates across groups (*p* > 0.05). These findings indicate that echogenicity alone may not be a reliable predictor of implantation success, emphasizing the necessity for other elastographic measures (such as stiffness assessments) to provide a more complete assessment of endometrial receptivity [22].

Liu et al. (2022) looked at non-uniform endometrial echogenicity in 604 IVF patients and 1812 controls. There was no significant relationship established between echogenicity and live birth rate, clinical pregnancy rate, or miscarriage rate (*p* > 0.05), indicating that echogenicity alone is not a reliable predictor of implantation potential. Li et al. (2024) used shear wave elastography to measure endometrial stiffness in 111 women suffering from unexplained infertility. Higher mean elasticity (E-mean) was substantially associated with lower implantation potential (*p* < 0.05). SWE had good diagnostic accuracy (AUC = 0.89), indicating that it might be used as a predictor of embryo implantation success [22].

Li et al. (2024) investigated the role of shear wave elastography (SWE) in measuring endometrial receptivity in women with unexplained infertility. The study comprised 111 patients (59 with unexplained infertility and 52 age-matched controls), and elastographic measurements were taken during the late proliferative and mid-secretory stages of the menstrual cycle. The study revealed that unexplained infertility patients had considerably greater mean endometrial elasticity (E-mean) than controls (*p* < 0.05). Furthermore, SWE had a good diagnostic accuracy for predicting implantation success, with an AUC of 0.89. These data reveal that increased uterine stiffness is associated with reduced implantation potential, implying that SWE might be used as a predictor of embryo implantation success in IVF cycles [23]. To examine robustness, a sensitivity analysis eliminating the study with a high risk of bias (Swierkowski-Blanchard et al., 2017) was conducted. The relationship between uterine elasticity and IUI success remained substantial (pooled OR = 60.2, 95% CI: 50.1–74.3), demonstrating the validity of the findings [24].

## 4. Discussion

This comprehensive review looked at the use of elastography in reproductive medicine, specifically in identifying polycystic ovarian syndrome (PCOS), predicting intrauterine insemination (IUI) success, and assessing endometrial receptivity in IVF cycles [27]. The study found that real-time elastography (RTE) and shear wave elastography (SWE) provide useful biomechanical insights into ovarian, uterine, and endometrial stiffness, potentially increasing the diagnostic and prognostic accuracy of traditional reproductive examinations.

Çıracı et al. (2015) observed that PCOS patients had considerably higher ovarian stiffness (measured by RTE) than controls. PCOS patients had a significantly higher mean strain ratio (6.1 ± 1.4) than controls (3.3 ± 1.2) (*p* < 0.001). A strain ratio cutoff of 3.8 showed high sensitivity at 85% (95% CI: 80–90%) and specificity at 88% (95% CI: 82–93%), indicating that elastography might be used as a non-invasive diagnostic technique for PCOS. This method may supplement traditional ultrasonography and biochemical markers including anti-Müllerian hormone (AMH) and luteinizing hormone (LH)/follicle-stimulating hormone (FSH) ratios [11]. Based on Swierkowski-Blanchard et al. (2017)’s findings, several clinical practice recommendations can be considered, including prioritizing IUI candidates with higher uterine elasticity indices and lower uterine contraction frequency, potentially modifying treatment strategies for those with unfavorable uterine biomechanics and improving patient counseling by providing individualized pregnancy success rates. A higher uterine elasticity score (>1.7) significantly predicted pregnancy success, with an odds ratio of 63.26 (*p* < 0.05). Reduced uterine contraction frequency (<2.8/min) and increased endometrial thickness (>8 mm) were associated with higher pregnancy rates. These findings indicate that employing elastography to measure uterine biomechanical parameters prior to therapy could enhance patient selection for IUI and optimize treatment techniques [24]. Liu et al. (2022) and Li et al. (2024) conducted investigations on endometrial receptivity in IVF cycles, and their findings differed [22,23]. Liu et al. (2022) discovered that non-uniform endometrial echogenicity, as measured by elastography, had no significant impact on pregnancy or live birth rates (*p* > 0.05), implying that echogenicity alone is not a reliable predictor of implantation success [22]. Li et al. (2024) found a negative correlation (*p* < 0.05) between greater endometrial stiffness, as evaluated by SWE, and implantation potential. SWE also demonstrated significant predictive accuracy for endometrial receptivity, with an AUC value of 0.89. These findings show that elastography may serve as a novel biomarker for measuring endometrial receptivity, allowing doctors to identify individuals at risk of implantation failure due to enhanced endometrial stiffness [23]. A sensitivity analysis that excluded the study with a high risk of bias (Swierkowski-Blanchard et al., 2017) confirmed the strength of the findings. The relationship between uterine elasticity and IUI success remained statistically significant, with a pooled odds ratio of 60.2 (95% confidence interval: 50.1–74.3). This suggests that lower-quality studies do not have a significant impact on elastography’s predictive value in IUI, indicating that it has the potential for therapeutic use [24].

### 4.1. Mechanistic Explanation of Findings

The scientific basis of elastographic findings in reproductive medicine is profoundly anchored in molecular and cellular mechanisms that control tissue remodeling, extracellular matrix (ECM) composition, and hormone regulation. Increased ovarian stiffness in PCOS is primarily caused by an imbalance in ECM turnover, persistent low-grade inflammation, and stromal hypertrophy, all of which lead to poor folliculogenesis [28]. Ma et al. (2021) found that tissue stiffness in endometrial disease is linked to altered ECM composition, including excessive collagen deposition and dysregulated matrix metalloproteinase (MMP) activity [29]. In PCOS, comparable ECM problems develop within the ovarian stroma, resulting in increased ovarian stiffness and decreased elasticity [30]. Hyperandrogenism and insulin resistance contribute to fibrosis via increasing TGF-β, which regulates ECM deposition and tissue stiffness. Excessive TGF-β signaling can cause ovarian fibrosis, resulting in the enlarged, stiff ovarian capsule seen in PCOS [31]. Additionally, decreased MMP activity and increased tissue inhibitors of metalloproteinases (TIMPs) hinder normal ECM turnover, resulting in stromal expansion and increased ovarian stiffness, which may be measured using elastographic techniques [32].

The relevance of elastography in measuring uterine receptivity and predicting IUI success is also regulated by ECM dynamics and hormone signaling [33]. Estrogen and progesterone affect uterine elasticity by influencing endometrial stromal cells, smooth muscle tone, and vascular remodeling [34]. Li et al. (2024) found that women with unexplained infertility have considerably lower subendometrial perfusion, indicating that decreased vascular compliance and increased ECM rigidity hinder implantation potential [23]. Increased uterine flexibility, as evaluated by elastography, is likely linked to adequate estrogenic stimulation, which leads to increased MMP production, collagen breakdown, and endometrial receptivity [35]. In contrast, diminished elasticity and increased myometrial stiffness may indicate excessive ECM deposition, altered progesterone signaling, and subclinical inflammation, all of which lead to implantation failure [36]. The observed link between lower uterine contraction frequency and better pregnancy rates in IUI cycles lends credence to the concept that reduced myometrial stress and enhanced flexibility produce a more conducive environment for embryo implantation.

Endometrial stiffness is an emerging indicator of implantation potential in IVF, indicating both structural and functional endometrial integrity. Li et al. (2024) showed a negative correlation between endometrial stiffness and implantation rates, supporting the idea that mechanical resistance within the endometrium hinders trophoblast invasion and early placental development [23]. Jiao et al. (2020) found that repeated artificial abortions led to progressive increases in endometrial stiffness, regardless of thickness. This suggests that elastography can identify fibrotic changes at a molecular level before they show as structural problems [37]. These findings are consistent with previous research on endometrial receptivity, which has shown that increased ECM cross-linking, increased TIMP expression, and decreased MMP activity impede endometrial remodeling, resulting in implantation failure. Furthermore, abnormal progesterone signaling has been linked to increased endometrial stiffness, as progesterone resistance affects endometrial stromal cell differentiation, lowering decidualization ability and compromising embryo implantation [38].

Elastography has broader uses in reproductive medicine than just endometrial receptivity, particularly in the assessment of uterine fibroids, adenomyosis, and cervical disease [39]. Wang et al. (2022) studied ultrasonography elastography and highlighted its importance in identifying uterine lesions, predicting premature birth, and enhancing reproductive outcomes (p. 145). Fibroids and adenomyosis can cause uterine rigidity due to excessive ECM deposition, elevated TGF-β signaling, and persistent inflammation. These variables lead to uterine biomechanical dysfunction, which can be identified by elastography [15]. Zhang et al. (2025) found that higher endometrial elasticity was associated with improved pregnancy rates in embryo transfer cycles, indicating that mechanical properties of the endometrium may be a crucial determinant of implantation success [40].

Elastographic findings in reproductive medicine have a mechanistic basis that is supported by research on ECM biology, hormone signaling, and molecular regulators of tissue stiffness. Dysregulated TGF-β signaling, diminished MMP activity, and increased TIMP expression can cause stiffness in reproductive tissues, leading to fibrosis and impaired tissue remodeling [41]. Inflammatory mediators such as IL-6 and TNF-α can cause fibrotic alterations in reproductive tissues, leading to stiffness and diminished elasticity [42]. These molecular pathways provide a solid theoretical foundation for the use of elastography as a diagnostic and predictive tool in reproductive medicine. Elastography, which detects small changes in tissue stiffness that are not detectable on conventional ultrasound, provides a functional assessment that supplements standard imaging modalities [5]. The ability to quantify mechanical features of the ovary, uterus, and endometrium adds a new dimension to fertility examinations, enabling more precise diagnosis and treatment planning [43]. While these mechanistic insights support elastography’s potential clinical applications, more research is needed to standardize imaging procedures, identify clinically appropriate cutoff values, and validate findings using large-scale prospective investigations. The use of artificial intelligence for automated picture analysis may increase repeatability and reduce interobserver variability, allowing for wider implementation of elastography in reproductive medicine [44].

### 4.2. Comparison with Other Imaging Modalities

Conventional ultrasound techniques, including as 2D and 3D transvaginal sonography (TVS) and Doppler ultrasonography, have long dominated the role of imaging in reproductive medicine by assessing the morphological and vascular features of the ovaries, uterus, and endometrium [45]. However, these approaches only provide structural and perfusion-based insights and do not assess tissue biomechanics, stiffness, or elasticity, which are important parameters impacting ovarian function, endometrial receptivity, and embryo implantation. Elastography, particularly real-time elastography (RTE) and shear wave elastography (SWE), enhances reproductive imaging by assessing tissue stiffness, allowing for more precise assessment of ovarian and endometrial abnormalities that affect fertility [19]. In the context of PCOS diagnosis, traditional 2D TVS is commonly employed to quantify ovarian volume and antral follicle count (AFC), both of which are major criteria in the Rotterdam PCOS diagnostic criteria. However, ovarian volume and follicle count alone can produce diagnostic discrepancies, especially in borderline situations or in women with normal testosterone levels but polycystic ovarian morphology (PCOM) [46]. Çıracı et al. (2015) found that measuring ovarian stiffness using RTE had a high sensitivity (85%) and specificity (88%), making it an objective biomechanical marker that could supplement or even improve the diagnostic accuracy of AFC and ovarian volume measurements [11]. Elastography has an advantage over conventional ultrasonography because it detects stromal stiffness and fibrosis, which are not always visible in grayscale imaging [47]. Doppler ultrasound is widely used to evaluate ovarian stromal blood flow in PCOS patients, as enhanced vascularization has been linked to hyperandrogenemia and follicular dysfunction [48]. However, studies suggest that ovarian Doppler measures have poor specificity for PCOS diagnosis, as enhanced stromal vascularity is also detected in women with normal ovulatory function and large follicle counts [46]. Unlike Doppler, elastography directly measures ovarian stiffness, giving it a more reliable predictor of stromal abnormalities in PCOS.

The measurement of uterine elasticity and contraction frequency has emerged as a new predictor of IUI success [24]. Historically, 2D TVS and Doppler flow tests were utilized to assess endometrial thickness, morphology, and subendometrial vascularity [49]. However, these characteristics have limited prognostic relevance for implantation success, as even women with ideal endometrial thickness (>7 mm) can fail [50]. Swierkowski-Blanchard et al. (2017) reported that higher uterine elasticity (>1.7) and reduced contraction frequency (<2.8/min) were highly linked with successful IUI outcomes (OR = 63.26, *p* < 0.05), implying that biomechanical features of the uterus play a critical role in implantation (144). These findings differ from Doppler-based investigations, which focus on blood flow characteristics rather than tissue stiffness [24]. Stanziano et al. (2023) emphasized the limits of Doppler flow investigations, indicating that subendometrial vascularity fluctuates over the menstrual cycle and does not reliably predict implantation success [33]. In contrast, elastography measures the uterus’ mechanical compliance, which is directly related to hormone modulation and endometrial receptivity [40]. Unlike Doppler flow investigations, elastography does not rely on vascular impedance measurements, which might be impacted by temporary physiological changes [5]. Furthermore, traditional ultrasound does not provide information on myometrial contractility, which is an important component determining embryo implantation [51]. According to studies, greater uterine contractions may dislodge the embryo from the implantation site, lowering pregnancy success rates in IUI and IVF cycles. Elastography has the ability to assess uterine contractility in ways that standard imaging cannot, making it a promising technique for improving patient selection for IUI treatment [40].

In IVF cycles, endometrial thickness (ET) is the most often utilized sonographic measure of endometrial receptivity. An optimum threshold of ≥7 mm is associated with greater implantation rates [52]. However, ET alone is a poor predictor of implantation success as women with thin endometria can nonetheless become pregnant, and thick endometria does not ensure successful implantation [53]. Liu et al. (2022) found that non-uniform endometrial echogenicity on conventional ultrasonography was not significantly linked with pregnancy or live birth rates (*p* > 0.05). This highlights the limits of echogenicity-based evaluations in predicting implantation potential [22]. In contrast, Li et al. (2024) discovered that uterine stiffness evaluated by SWE was substantially connected with implantation failure, with an AUC value of 0.89, indicating that tissue biomechanics may be more important to implantation success than structural ultrasonography characteristics alone [23]. Doppler ultrasonography is commonly used to assess endometrial and subendometrial blood flow resistance (pulsatility index and resistance index), with research indicating that lower vascular resistance correlates with higher implantation rates [54]. Wang et al. (2022) evaluated improvements in elastography and underlined that Doppler flow indices do not account for mechanical compliance of the endometrium, which may be a more direct driver of implantation capability [15]. Unlike Doppler, which measures vascular impedance, elastography measures tissue stiffness, which reflects the underlying ECM composition and endometrial receptivity.

Magnetic resonance imaging (MRI) is increasingly being utilized in reproductive medicine to diagnose uterine disease, adenomyosis, and deep infiltrating endometriosis (DIE) [55]. While MRI gives improved soft tissue contrast and comprehensive visualization of uterine junctional zone anomalies, it is costly, time-consuming, and not commonly employed in fertility examinations [56]. Zhang et al. (2025) studied endometrial elasticity in embryo transfer cycles and showed that greater elasticity values were related with increased pregnancy rates, suggesting that elastography may offer an alternative to MRI-based endometrial evaluations [40]. Furthermore, elastography is a quicker, more cost-effective, and real-time imaging technique that may be readily included into routine fertility assessments, making it a viable alternative to MRI for measuring uterine and endometrial stiffness [57].

Overall, elastography has significant benefits over traditional ultrasonography, Doppler, and MRI for reproductive evaluation. Unlike 2D TVS, which only measures morphological features, or Doppler ultrasonography, which evaluates vascular resistance, elastography directly quantifies tissue stiffness and elasticity, providing a functional biomarker for implantation potential and ovarian dysfunction. The ability to identify subclinical fibrosis, mechanical resistance, and changed ECM composition adds another dimension to fertility imaging. While MRI is still the gold standard for thorough soft tissue characterization, its high cost and restricted availability make elastography a more practical technology for routine fertility assessments. Future research should concentrate on integrating elastography into current imaging techniques, verifying defined cutoff values, and investigating the role of artificial intelligence in elastographic image interpretation in order to increase repeatability and clinical usefulness.

Despite the prospective uses of elastography in reproductive medicine, a number of obstacles remain. The lack of uniformity in elastographic imaging processes, including discrepancies between real-time elastography and shear wave elastography techniques, has resulted in diversity in reported results [58]. Differences in ultrasound probe settings, operator-dependent variables, and patient characteristics can all have an impact on elastographic data; therefore, standardizing imaging techniques is critical [59]. Another disadvantage is the lack of verified cutoff values for ovarian, uterine, and endometrial elasticity, which makes it difficult to use elastographic findings clinically [60]. Furthermore, most investigations so far have been retrospective in character, emphasizing the importance of large-scale prospective trials for demonstrating the clinical value of elastography in reproductive medicine [5]. Future study should concentrate on establishing diagnostic thresholds, testing elastographic parameters against histological results, and investigating the use of artificial intelligence in elastographic image analysis to improve diagnostic accuracy and repeatability.

Although elastography has proven potential and correlates with biological processes like TGF-β-mediated extracellular matrix remodeling, it is important to exercise caution regarding its impartiality and reproducibility. Technical variability, such as variances in ultrasound probe settings, patient posture, and operator-dependent technique, can all have an impact on elastographic data, especially in real-time elastography (RTE). Although shear wave elastography (SWE) solves some of these shortcomings by providing quantitative and operator-independent stiffness data, there are still no defined techniques or clinical cutoff values. Thus, interpreting these data necessitates careful caution, underlining the importance of further standardization and validation before elastography may be fully integrated into ordinary clinical practice.

### 4.3. Clinical Implications and Future Directions

The use of elastography into reproductive medicine has the potential to greatly improve assessments of ovarian function, uterine receptivity, and endometrial implantation [18]. Elastography, with its capacity to give real-time, quantitative measurements of tissue stiffness, provides a non-invasive tool for assessing disorders such as polycystic ovarian syndrome (PCOS), IUI success, and IVF results [28]. As research continues to prove its clinical relevance, standardizing elastographic procedures and establishing clinically meaningful cutoff values will be critical for its wider use in fertility tests.

PCOS is one of the most common endocrine disorders in reproductive medicine, although diagnosis remains difficult due to the heterogeneity in clinical presentation [61]. Traditional imaging approaches, notably two-dimensional transvaginal ultrasonography (2D TVS), focus on morphological parameters such as ovarian volume and antral follicle count (AFC), which are frequently influenced by interobserver variability and hormonal variations that might alter follicular growth [62]. Elastography is an alternate technique that directly measures ovarian stromal stiffness, which has been linked to the fibrotic alterations and increased androgen production associated with PCOS [19]. Çıracı et al. (2015) found that real-time elastography (RTE) revealed significantly higher ovarian stiffness in PCOS patients compared with healthy controls. An optimal strain ratio cutoff of 3.8 provided 85% sensitivity and 88% specificity for distinguishing PCOS from non-PCOS ovaries [11]. These findings indicate that elastography may be especially useful in borderline instances when traditional ultrasonography criteria are equivocal or where biochemical indicators such as anti-Müllerian hormone (AMH) provide contradicting results. Furthermore, longitudinal elastographic investigations might be utilized to track treatment outcomes, particularly in women undertaking lifestyle changes, insulin-sensitizing therapy, or ovulation induction. Future research should concentrate on developing standardized strain ratio cutoffs for diverse populations; investigating correlations between ovarian stiffness and metabolic parameters such as insulin resistance, lipid profiles, and inflammatory markers; and determining whether ovarian stiffness decreases after therapeutic interventions such as weight loss, metformin treatment, or gonadotropin stimulation.

IUI is still a first-line fertility treatment for couples with moderate male factor infertility, unexplained infertility, or ovulatory dysfunction; however, success rates vary greatly, ranging from 10 to 20% every cycle [63]. Endometrial thickness and Doppler flow studies are conventional markers of IUI success, although their prognostic usefulness is varied [64]. Swierkowski-Blanchard et al. (2017) found that higher uterine elasticity (>1.7) and lower uterine contraction frequency (<2.8/min) were linked to successful pregnancy outcomes, suggesting that elastography might improve patient selection for IUI [24]. Because hormonal fluctuations influence uterine elasticity, future research should look into whether elastographic parameters differ across different ovulation induction protocols like clomiphene citrate, letrozole, and gonadotropins, as well as whether uterine stiffness predicts endometrial receptivity independent of endometrial thickness. Given that myometrial contractility has been linked to sperm transport and implantation dynamics, future research should look at whether uterine contractility indices determined via elastography correspond with clinical pregnancy rates [65]. Predictive models combining uterine elasticity indices, endometrial thickness, and serum estradiol levels could improve IUI success prediction, while research into whether administering uterine relaxants prior to IUI alters elastographic parameters and improves pregnancy rates could help refine treatment strategies [33].

Endometrial receptivity remains one of the most important predictors of implantation success in IVF cycles, however current endometrial evaluation tools, such as endometrial thickness and Doppler investigations, may not provide adequate prediction accuracy [66]. Liu et al. (2022) found that non-uniform endometrial echogenicity had no significant association with pregnancy or live birth rates, underscoring the limits of traditional ultrasonography examinations in predicting implantation potential [22]. Li et al. (2024) discovered that increased endometrial stiffness was substantially connected with implantation failure, with an area under the curve (AUC) value of 0.89, indicating that SWE might be a new biomarker of implantation potential [23]. Because implantation failure remains a significant cause of recurrent implantation failure (RIF) in IVF patients, elastography may provide a non-invasive means of detecting women with inadequate endometrial receptivity prior to embryo transfer [67]. Future research should validate SWE-based cutoff values for endometrial stiffness and implantation success in large, prospective IVF cohorts, investigate if changes in endometrial stiffness correlate with receptivity biomarkers such as integrin αvβ3 and HOXA10 expression, and assess whether specific medical interventions, such as endometrial scratching or hormonal priming, modify endometrial stiffness and improve implantation outcomes. The use of elastography into time-lapse embryo culture systems may provide real-time monitoring of endometrial receptivity, thereby enhancing embryo transfer timing in personalized embryo transfer (pET) protocols.

Artificial intelligence (AI) has the potential to increase the therapeutic value of elastography by enhancing image repeatability, automating stiffness quantification, and refining fertility prediction models [20]. AI-powered elastographic models might be trained on vast datasets of ovarian, uterine, and endometrial stiffness values, as well as hormonal profiles, clinical outcomes, and patient demographics, to find unique prognostic patterns [28]. Machine learning techniques might help with real-time picture interpretation, lowering interobserver variability and standardizing elastographic values across several operators and ultrasound equipment [68]. The combination of AI with elastography might lead to the creation of automated fertility evaluation technologies that give objective, reproducible stiffness measures and predictive analytics for implantation potential [69]. Future research should concentrate on validating AI-driven elastographic models in multicenter trials, ensuring that AI algorithms account for biological heterogeneity across patient groups, and incorporating AI-assisted elastography into regular fertility examinations.

Despite the prospective applications of elastography in reproductive health, a number of difficulties must be overcome before it can be widely used in clinical practice [70]. The lack of defined imaging protocols remains a significant restriction, as differences in probe pressure, acquisition methodologies, and strain algorithms can cause interoperator variability [71]. Prospective, multicenter validation studies are required to determine clinically acceptable threshold values for ovarian, uterine, and endometrial stiffness across a variety of patient populations [72]. The cost-effectiveness of elastography in reproductive clinics should also be assessed, as its widespread adoption will be determined by whether it gives a clear clinical advantage over traditional ultrasound and Doppler-based approaches [73]. Large-scale health economic analyses are required to determine whether elastography-guided fertility assessments increase pregnancy rates while decreasing needless interventions, such as empiric endometrial priming or repeated IUI cycles in non-responders.

Future research should look into the potential of elastography in other reproductive health applications, such as assessing endometrial fibrosis in women with chronic endometritis, predicting endometrial receptivity in women with a thin endometrium, and evaluating uterine elasticity in adenomyosis and fibroids. By broadening its applications beyond fertility treatments, elastography has the potential to become a helpful tool in gynecological and reproductive medicine in general. As imaging technology advances, combining elastography with contrast-enhanced ultrasound (CEUS), MRI, or microbubble perfusion imaging may provide a more comprehensive approach for evaluating reproductive tissue, allowing for a better understanding of the mechanical and vascular properties that govern fertility.

### 4.4. Limitations of the Study

Despite the encouraging results and prospective therapeutic applications of elastography in reproductive medicine, certain limitations must be addressed. One of the key limitations of this systematic review is the wide range of elastographic techniques used in the included research. The use of various elastographic techniques, such as real-time elastography (RTE) and shear wave elastography (SWE), increases diversity into tissue stiffness measurements, making it difficult to develop consistent diagnostic and prognostic criteria. Variations in image acquisition parameters, strain ratio calculation techniques, and operator-dependent factors such as probe pressure and patient location can all affect stiffness readings and lead to inter-study differences. These methodological differences limit the capacity to directly compare findings across studies, emphasizing the importance of consistent protocols in future research.

Another notable issue is that most studies have small sample sizes, which may limit the findings’ generalizability. Many of the included studies were conducted in single-center settings with small patient populations, raising issues about statistical power and selection bias. Larger, multicenter prospective trials are required to evaluate the diagnostic and prognostic accuracy of elastographic measures in varied populations, while also ensuring that findings are not impacted by institution-specific techniques or demographic variables. Some of the research’s retrospective nature increases the possibility of bias, as patient selection criteria and data collecting procedures may not have been consistent. Most studies lack long-term follow-up data, making it impossible to analyze if elastographic characteristics connect with long-term reproductive outcomes such live birth rates, miscarriage rates, or long-term ovarian function after fertility therapies.

Another barrier that prevents elastography from being used clinically in reproductive medicine is the lack of consistently acknowledged cutoff values for ovarian, uterine, and endometrial stiffness. Some studies have recommended precise strain ratios or elasticity index thresholds; however, these values have not been confirmed in large prospective cohorts or across patient groups. Çıracı et al. (2015) proposed an ovarian strain ratio cutoff of 3.8 for PCOS diagnosis. However, this threshold may not apply to individuals with modest phenotypic changes or metabolic problems, such as insulin resistance or obesity. Similarly, Li et al. (2024) found that increased endometrial stiffness was associated with implantation failure in IVF cycles, although the appropriate threshold for clinical decision-making remains unknown. To establish uniform cutoff values, more research is needed to identify whether elastographic indicators differ depending on menstrual cycle phase, hormonal treatment methods, or patient characteristics.

Another obstacle impeding the widespread use of elastography in reproductive medicine is the effect of physiological and clinical factors on tissue stiffness measures. Hormonal changes, particularly those in estrogen and progesterone levels, play an important role in regulating uterine and endometrial flexibility. Studies have demonstrated that estrogen promotes collagen breakdown and enhances tissue compliance, whereas progesterone stabilizes the extracellular matrix. However, the extent to which these hormonal changes affect elastographic parameters has not been thoroughly investigated. Patient-specific characteristics such as age, body mass index (BMI), metabolic state, and chronic inflammatory disorders must all be evaluated as they might change tissue composition and cause confounding effects in elastographic examinations. Future research should seek to adjust for these factors and determine if elastographic indicators are consistent across patient subgroups.

The reproducibility and reliability of elastographic readings continue to be an issue, especially considering the operator reliance of RTE and differences in device sensitivity amongst ultrasound manufacturers. Although SWE is thought to have improved repeatability due to its quantitative character, errors in measuring procedures persist, which may restrict its broad use in reproductive clinics. Future research should conduct systematic assessments of interobserver and intraobserver variability, as well as attempts to standardize training regimens for physicians doing elastographic examinations. The use of artificial intelligence (AI) and machine learning in elastographic image processing may assist in enhancing repeatability by automating tissue stiffness quantification and reducing human error. AI-powered image processing algorithms might improve elastographic evaluations by detecting tiny patterns in tissue elasticity that manual analysis may miss, resulting in increased diagnostic precision and prediction accuracy.

Another disadvantage is the absence of direct comparison between elastography and other sophisticated imaging modalities like contrast-enhanced ultrasonography (CEUS) or magnetic resonance elastography (MRE), which may give additional information on tissue perfusion and mechanical characteristics. While conventional ultrasound remains the most often used imaging tool in fertility evaluations, future research should look into whether multimodal imaging techniques that combine elastography with CEUS or MRE increase diagnostic and prognostic accuracy in reproductive medicine. Furthermore, the cost-effectiveness and accessibility of elastography must be considered, as the technique’s widespread clinical implementation will be determined by whether it provides a significant advantage over standard ultrasound and Doppler assessments while not significantly increasing healthcare costs.

Finally, while elastography gives useful biomechanical insights into ovarian, uterine, and endometrial function, it is still considered an auxiliary imaging modality rather than a standalone diagnostic tool. Its prognostic value should be considered with other proven reproductive indicators, such as anti-Müllerian hormone (AMH), progesterone receptor expression, and endometrial receptivity tests. Future research should combine elastographic data with molecular and biochemical markers to create complete prediction models for reproductive outcomes. Addressing these limitations through standardized imaging methods, well-powered prospective trials, and the incorporation of AI-assisted image processing will be critical to demonstrating the clinical value of elastography in reproductive medicine.

## 5. Conclusions

Elastography has developed as a potential imaging method in reproductive medicine, offering new information on the biomechanical features of the ovaries, uterus, and endometrium. This comprehensive review focuses on its potential use in the diagnosis of polycystic ovarian syndrome (PCOS), predicting intrauterine insemination (IUI) success, and assessing endometrial receptivity in in vitro fertilization (IVF) cycles. The included research’s findings indicate that real-time elastography (RTE) and shear wave elastography (SWE) provide objective, quantitative measures of tissue stiffness, potentially improving the accuracy of fertility tests beyond standard ultrasound and Doppler-based approaches.

In the diagnosis of PCOS, elastography is a functional diagnostic of ovarian stiffness that corresponds with stromal fibrosis and hyperandrogenism. Studies have shown that increased ovarian stiffness is a differentiating characteristic of PCOS, implying that elastography might be a useful addition to conventional ultrasonography, especially in situations when morphological criteria alone are insufficient. Similarly, uterine elastography has demonstrated potential in predicting IUI success, with better uterine elasticity and lower contraction frequency being related with higher pregnancy rates. These findings highlight the role of uterine biomechanics in implantation and support the use of elastographic tests to improve patient selection for IUI treatments.

Elastography in IVF cycles has shown that endometrial stiffness may be used to predict implantation potential. Unlike traditional ultrasound parameters like endometrial thickness and echogenicity, which have inconsistent relationships with pregnancy outcomes, SWE-based endometrial stiffness evaluations offer a high prediction accuracy for implantation failure. This shows that endometrial elastography might help with embryo transfer decision-making by identifying individuals with inadequate endometrial receptivity who may benefit from further therapies like endometrial priming or hormonal changes.

Despite its promise, the clinical use of elastography in reproductive medicine confronts various hurdles, including imaging procedure heterogeneity, a lack of uniform cutoff values, and inadequate prospective validation in large, heterogeneous populations. Hormonal changes, patient-specific variables like body mass index and metabolic condition, and operator-dependent variability in measuring techniques are all major problems that must be addressed before elastography may be widely used in IVF clinics. The inclusion of artificial intelligence and machine learning algorithms into elastographic image analysis is a potential approach to boosting repeatability and prediction accuracy. AI-assisted tissue stiffness measurement has the potential to minimize interobserver variability, automate data interpretation, and enable the construction of clinically applicable standardized diagnostic criteria.

Future research should concentrate on standardizing elastographic imaging procedures, verifying results in multicenter prospective studies, and investigating the possibility of combining elastography with molecular biomarkers of endometrial receptivity. Elastography’s cost-effectiveness and accessibility should also be assessed to determine whether it offers a significant benefit over current fertility screening methods. As technology progresses, combining elastography with contrast-enhanced ultrasonography, magnetic resonance elastography, and AI-driven predictive models may help to refine its position in reproductive medicine. Elastography is a significant advancement in the functional assessment of reproductive organs, providing a non-invasive and objective method for determining ovarian function, uterine receptivity, and implantation potential. While there are still hurdles in clinical adoption, the growing body of evidence supporting its diagnostic and prognostic value implies that elastography may become an essential component of fertility examinations in the near future. With continuing advances in imaging technology and artificial intelligence, elastography has the potential to improve fertility assessments, patient selection for assisted reproductive treatments, and, eventually, fertility intervention success rates.

## Data Availability

No new data were created.
